# Rolling circle amplification of synthetic DNA accelerates biocatalytic determination of enzyme activity relative to conventional methods

**DOI:** 10.1038/s41598-020-67307-9

**Published:** 2020-06-24

**Authors:** Timin Hadi, Nicole Nozzi, Joel O. Melby, Wei Gao, Douglas E. Fuerst, Erik Kvam

**Affiliations:** 1 GlaxoSmithKline, 1250 South Collegeville Road, Collegeville, Pennsylvania 19426 USA; 2 GE Global Research, One Research Circle, Niskayuna, NY 12309 USA

**Keywords:** Expression systems, Biosynthesis

## Abstract

The ability to quickly and easily assess the activity of large collections of enzymes for a desired substrate holds great promise in the field of biocatalysis. Cell-free synthesis, although not practically amenable for large-scale enzyme production, provides a way to accelerate the timeline for screening enzyme candidates using small-scale reactions. However, because cell-free enzyme synthesis requires a considerable amount of template DNA, the preparation of high-quality DNA “parts” in large quantities represents a costly and rate-limiting prerequisite for high throughput screening. Based on time-cost analysis and comparative activity data, a cell-free workflow using synthetic DNA minicircles and rolling circle amplification enables comparable biocatalytic activity to cell-based workflows in almost half the time. We demonstrate this capability using a panel of sequences from the carbon-nitrogen hydrolase superfamily that represent possible green catalysts for synthesizing small molecules with less waste compared to traditional industrial chemistry. This method provides a new alternative to more cumbersome plasmid- or PCR-based protein expression workflows and should be amenable to automation for accelerating enzyme screening in industrial applications.

## Introduction

Industrial adoption of biocatalytic transformations for the syntheses of fine chemical intermediates has increased over the last decade, in part because lowered costs of DNA sequencing and synthesis have enabled the acquisition and characterization of novel enzyme classes at a rapid pace^1^. Moreover, methods for mutating and evolving enzymes toward specific applications have facilitated biocatalytic reactions that were previously inaccessible.

A common practice for identifying biocatalytic starting-points is to screen collections of enzymes in a high-throughput manner to identify promising activity. After initial candidates are identified, biochemical properties including enzyme catalytic rate, stereoselectivity, and stability are typically optimized. If further improvements are desired to meet specific process requirements, enzymes may be modified over several rounds of directed evolution^2^. Screening panels are typically 96-well collections of recombinant enzymes produced in a convenient expression host (usually *E. coli*). This form of cell-based production is easily adapted to automated workflows, but new strategies for speeding progress and reducing costs of promising “hits” are increasingly desired^1,3^. Cell-free protein synthesis (CFPS) is one technique that has been explored to accelerate the process of identifying enzymes for industrial applications^4^.

Cell-free synthetic biology requires substantial amounts of template DNA to express one or more RNA and/or protein targets of interest; consequently, DNA is one of the most expensive reagents in cell-free reactions^5^. Template DNA is traditionally prepared as either circular plasmid or synthetic linear DNA fragments depending on whether the cell-free system contains nucleases that degrade linear DNA^6,7^. Since plasmids are laborious and time-consuming to design and propagate, synthetic linear DNA fragments have emerged as preferred templates in synthetic biology for faster productivity and cycling times. However, methods of scaling linear DNA “parts” for cell-free systems almost exclusively rely on polymerase chain reaction (PCR), which can be burdensome for three reasons: (1) specific primer sequences must be designed and obtained prior to amplification, (2) “jackpot” mutations can occur during amplification to reduce the quality of the DNA amplicon pool^8^, and (3) volumetric scale-up of the thermocycling reaction is often challenging and post-PCR purification steps are required for high-yield expression in cell-free systems^9^. More importantly, PCR amplicons are unstable in nuclease-containing cell extracts unless subsequently converted into closed-circular DNA^10,11^, or synthesized with long protective 5′/3′ extensions^12^, or coordinated with exonuclease inhibitors^13^.

Here, we introduce an alternative method for amplifying synthetic linear DNA “parts” and demonstrate ease-of-use for screening enzyme activity and substrate preferences (cell-free) with comparable predictive power to conventional production in living cells. This cloning-free method involves amplifying DNA fragments as minicircles via random-primed rolling circle amplification (RCA) to generate high concentrations of concatemerized DNA with little risk for jackpot error propagation^14,15^. When the resulting unpurified RCA product is incubated with cell-free extract, the overall time and labor required for recombinant *E. coli* expression of an enzyme panel for biocatalytic chemical transformations is reduced, thereby enabling faster “first pass” characterization of enzyme-substrate activity compared to conventional cellular expression. We envision minicircle RCA to be suitable for automated/robotic platforms to advance cell-free synthetic biology using synthetic DNA fragments.

## Results

### Holistic comparison of workflow cycle time for protein expression

**Figure 1:**
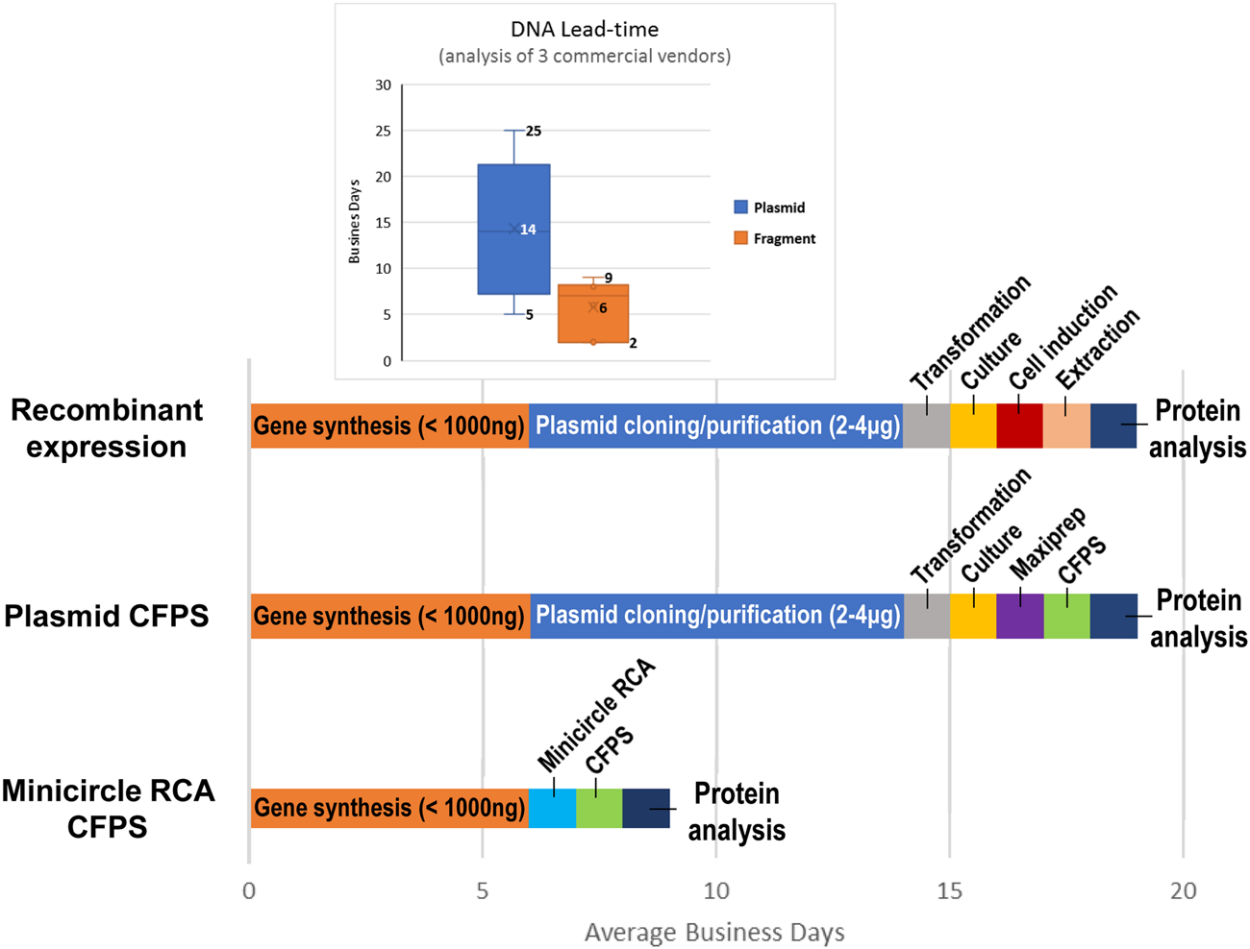
Summary of average cycle time for industrial enzyme screening using different DNA propagation and expression techniques.

The time and labor required to obtain DNA is often ignored when analyzing the productivity of cell-free synthetic biology. For example, while protein may be rapidly expressed in less than 24 hours via cell-free protein synthesis, the DNA template encoding the protein-of-interest often takes many days to convert from a digital sequence into physical DNA before protein expression can occur. We gathered commercial lead-time estimates (as advertised) for sequence-verified plasmids and synthetic DNA fragments from three leading providers. Lead-time for DNA fragment synthesis is considerably faster (by a net range of 3–16 business days) than plasmids (Fig. 1, inset). Because commercial estimates vary as a function of the size and complexity of a desired DNA sequence, we used the mathematical average (14 days for plasmid, 6 days for DNA fragments) to compare holistic cycle-times for protein expression, starting from a digital sequence and ending with functional protein (Fig. 1). In this context, fragment-based CFPS is at least 50% faster and encompasses fewer labor tasks than either plasmid-based expression in *E. coli* or plasmid-based CFPS, thereby accelerating protein characterization within a shorter period (Fig. 1). To satisfy DNA input requirements for CFPS (without using PCR), we scaled-up linear DNA fragments using minicircle RCA, in which as few as picograms of linear DNA are ligated into a minicircle for subsequent rolling-circle amplification using random primers. We subsequently validated the time and labor savings of minicircle RCA for screening a panel of enzyme sequences relative to conventional plasmid-based expression, as outlined below.

### Selection and acquisition of enzyme cDNA

**Figure 2:**
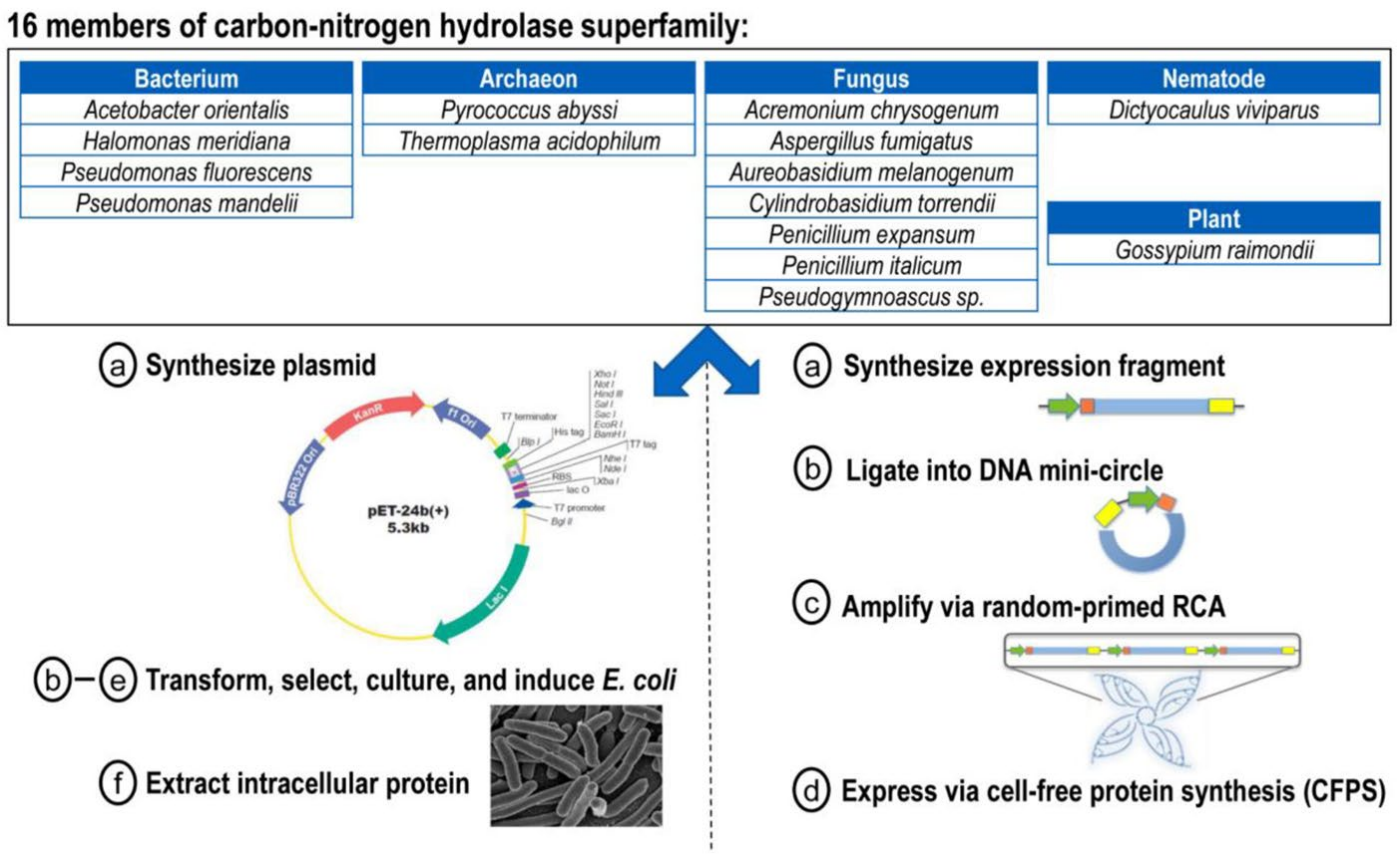
Biocatalytic study design for comparative cell-based and cell-free protein synthesis of 16 cDNA sequences from the carbon-nitrogen hydrolase superfamily.

To demonstrate industrial utility, we selected 16 cDNA sequences from the carbon-nitrogen hydrolase superfamily spanning five different evolutionary kingdoms and compared enzymatic activity following synthetic or conventional recombinant expression (Fig. 2). Enzymes from this superfamily, when coupled with novel protein engineering techniques, offer the potential to synthesize small-molecule drugs faster with less waste compared to traditional medicinal chemistry^16^. To obtain conventional plasmids, cDNA sequences were submitted to Genscript for delivery in a standard expression vector (pET-24b). For linear DNA fragment synthesis, cDNA sequences were submitted to IDT for delivery as gBlocks encoding similar 5′ and 3′ UTR regions as pET-24b plasmid but lacking the lac operator sequence. Coding regions for each gBlock were optimized for cell-free expression using a GE-proprietary algorithm that re-codes problematic sequence-based motifs, such as those affecting ribosome processing and T7 RNA polymerase^17^. Unique BamHI and BglII restriction sites were designed into each gBlock at the penultimate 5′ and 3′ ends, respectively, to mediate intramolecular ligation. While DNA plasmids were transformed into different *E. coli* strains for plasmid maintenance and protein expression, respectively, gBlocks were amplified by minicircle RCA as outlined in Fig. 2 and described in Methods section. Overall, RCA generated an average yield of 27 µg of amplified DNA (±4.8 µg, see Supplementary Table S1) per 100 µl reaction, thereby yielding sufficient template concentration (average 271 ng/µL, unpurified) for CFPS in a commercial *E. coli* lysate (Expressway, Thermo Fisher Scientific). Unlike published RCA-enabled workflows reported by others^18^, our minicircle amplification strategy involves no intermediate PCR scale-up, thereby eliminating the PCR workflow complications summarized in the preceding section.

### Comparison of enzymatic activity by different screening workflows

**Figure 3:**
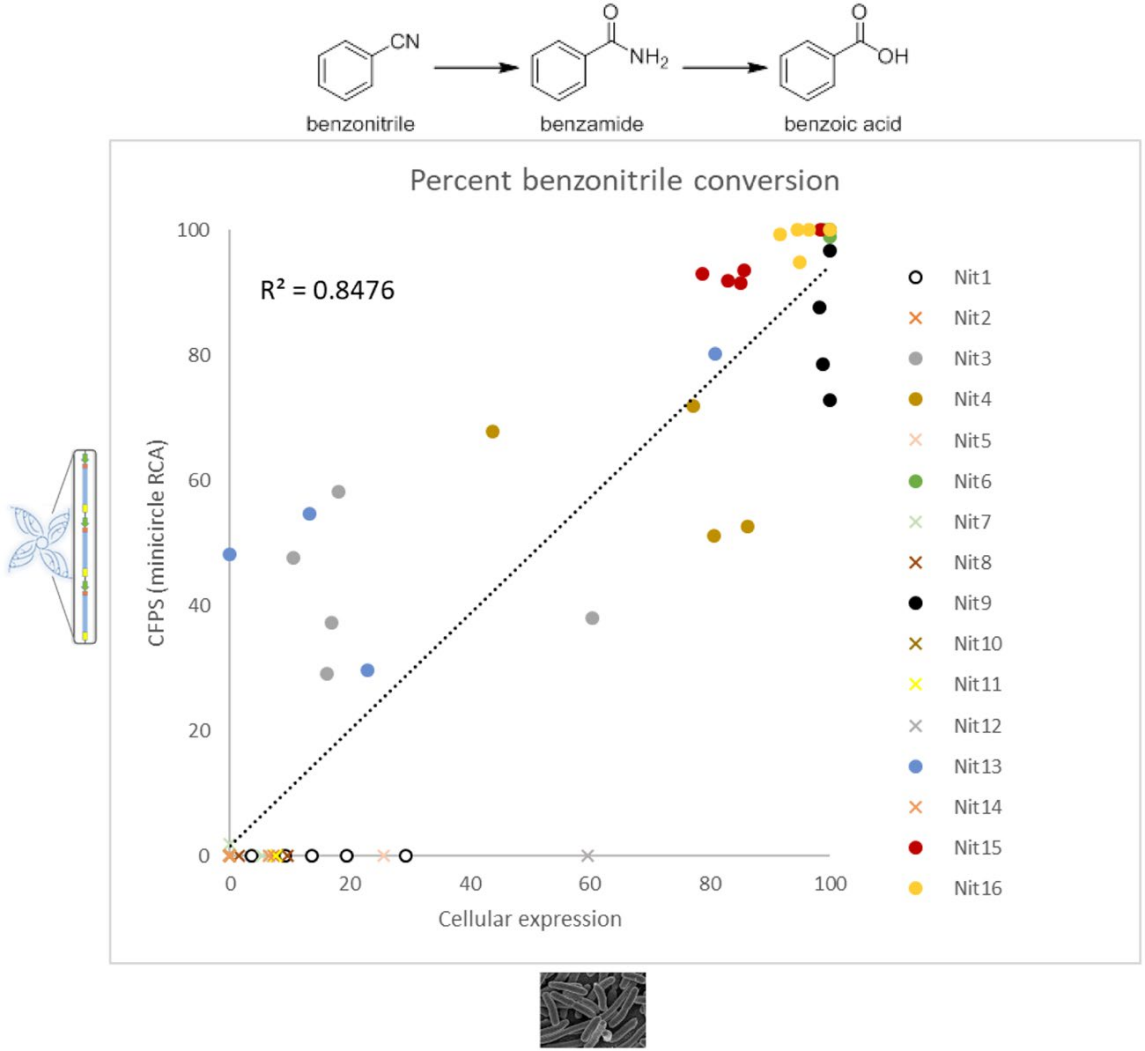
Comparison of benzonitrile hydrolysis activity across 16 putative nitrilases produced using either minicircle RCA-enabled CFPS or *E. coli*-based cellular expression. Raw data are plotted from at least 4 experimental tests, and data were collected by different lab operators.

We compared cellular vs. cell-free enzymatic activity for 16 putative nitrilases in a typical industrial enzyme screening workflow. For cell-based expression, we transformed pET-24b plasmids into *E. coli* BL21 cells to overexpress enzymes of interest, followed by cell lysis and substrate conversion testing in crude cell extract (analogous to published high-throughput methods^19^). For cell-free synthesis, we expressed putative nitrilases by minicircle RCA-enabled CFPS. We began our enzyme characterization by testing the conversion of benzonitrile (a common aromatic nitrile) to benzoic acid. Comparative high performance liquid chromatography (HPLC) of benzoic acid formation from the 16 tested enzymes revealed a favorable correlation coefficient (R^2^ of 0.8476) between cell-based expression and minicircle RCA-enabled CFPS (Fig. 3). Because these experiments were performed by three different individuals, inter-operator variability and the stochasticity of cell-based expression likely contributed to experimental variance among the replicate tests depicted in Fig. 3. Initial CFPS control reactions using pET-24b plasmid showed high activity dropout (likely from lac operator-mediated transcriptional repression) so we re-cloned each of the 16 nitrilases from pET-24b into pEXP5 plasmid (as recommended by the extract manufacturer). We then propagated the pEXP5 plasmids by rolling circle amplification^20^ and compared CFPS nitrilase activity against minicircle RCA-enabled CFPS. This cell-free comparison revealed similar benzonitrile hydrolysis activities between plasmid RCA and minicircle RCA, although Nit3 and Nit13 exhibited higher catalytic activity as minicircle RCA than plasmid RCA (see Supplementary Fig. S1). This finding is likely a consequence of the propriety sequence optimization process we performed on minicircle DNA but not plasmids. We did not examine these differences further since plasmid-based CFPS workflows greatly increase the cycle time for screening (Fig. 1).

We next repeated minicircle RCA-enabled CFPS reactions with a non-normalized amounts of template input (i.e. a fixed volume such that each CFPS reaction contained a variable amount of RCA, rather than exactly 500 ng). Gratifyingly, nitrilase activity did not significantly differ between normalized and non-normalized CFPS reactions (R^2^ of 0.98, see Supplementary Fig. S2), although none of these reactions contained DNA below the recommended template concentration for the ExpressWay system (10 ng per μL of reaction volume). Nevertheless, we believe that pre-analytical DNA quantitation and normalization steps, although achievable and potentially automatable, are undesirable when attempting to screen large numbers of enzymes by CFPS.

### Comparison of enzymatic substrate specificity

**Figure 4:**
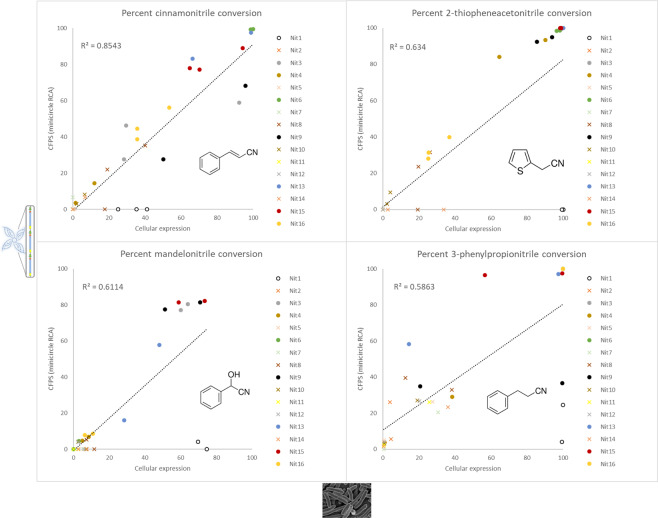
Comparison of substrate hydrolysis activity using cinnamonitrile, 2-thiopheneacetonitrile, mandelonitrile, or 3-phenylpropionitrile with 16 nitrilases produced using either minicircle RCA-enabled CFPS or *E. coli*-based cellular expression. Raw data are plotted from at least 2 independent experimental tests, which were performed by different lab operators.

**Figure 5:**
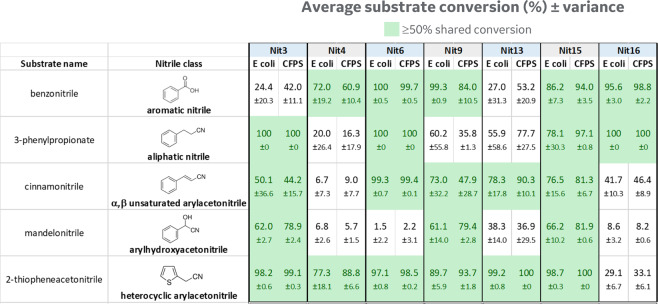
Agreement in substrate conversion results for select nitrilase “hits”. Table depicts the mathematical average activity (±s.d.) from the raw data plotted in Figs. 3 and 4. Enzymes showing ≥50% substrate conversion with both minicircle RCA-enabled CFPS and *E. coli* cell-based expression are shaded in green. For benzonitrile conversion (n ≥ 4 replicates), differences between means are not statistically significant by one-way ANOVA, except for Nit 9 (p < 0.05). Similarly, no significant differences are observed between means for cinnamonitrile and 2-thiopheneacetonitrile conversion when at least n = 3 replicates were tested (i.e. Nit 3, Nit 15, and Nit 16).

To further test minicircle RCA-enabled CFPS for substrate screening, we extended our activity studies to four additional nitriles: 3-phenylpropionitrile, mandelonitrile, 2-thiopheneacetonitrile, and cinnamonitrile. Although some of the resulting correlation coefficients between cell-based and CFPS screening appeared less proportional with these substrates compared to benzonitrile (R^2^ of 0.59, 0.61, 0.63 and 0.85, respectively, vs 0.85 for benzonitrile), qualitative activity trends were strong for all tested enzymes except Nit1 (Fig. 4, open circles). Overall “hit” rates (defined as >25% conversion to hydrolyzed product among the 16 enzymes analyzed) were between 31–62% for cell-based assays and 22–62% for CFPS assays depending on the tested chemical substrate. The observed discordance in Nit1 activity between cell-based and CFPS expression is unclear and warrants further investigation into possible inhibitory substances from the CFPS reaction, or perhaps lack of enzyme post-translational modification or accessory proteins that might be critical for this under-studied enzyme. For all other positive enzyme “hits,” the substrate patterns that emerged from our comparative analysis revealed similar nitrilase taxonomies^21^ (Fig. 5). For example, we observed that *Pseudomonas fluorescens* NitA (Nit3 in our study) exhibited both arylacetonitrilase and aliphatic nitrilase activities, confirming previous reports^22^.

## Discussion

In summary, this systematic comparison demonstrates that minicircle RCA-enabled synthetic biology enables comparable enzyme activity to plasmid- and cell-based workflows in almost half the time. When attempting to identify enzymes that catalyze specific reactions, a wide net is generally cast to increase the chances of acquiring useful diversity. Although computational techniques have increased the ‘hit’ rate of this process^1^, a relatively high number of dropouts are still typically observed. For cell-based screening workflows, enzymes are typically ordered as plasmid constructs and taken through expression and screening workflows (Fig. 1). Enzymes that have promising activity will be examined in greater detail while dropouts await secondary assays to test if some activity can be detected. At this stage, the reason for a putative enzyme becoming a drop-out will usually remain unknown, but often is the result of poor overexpression, improper protein folding, instability, or toxicity during cell growth and expression. When screening large numbers of putative enzymes, the time and effort required to individually troubleshoot and confirm niche reasons behind a dropout is generally not deemed worth the investment; that several potential enzymes will be ‘missed’ becomes an acceptable risk that can only be mitigated by carefully examining each enzyme individually. In this regard, any drop-outs from CFPS-based screening would be subject to a similar fate.

Replacing cell-based expression with CFPS for enzyme discovery and screening is not without some uncertainty. The same issues that influence cell-based activity drop-outs are also possible with CFPS (except for toxicity-based concerns). Certain types of enzymes may not be amenable to CFPS screening, for instance, proteins requiring significant post-translational modifications are poor candidates for *E. coli* lysate-based CFPS^23^. Complex protein-folding or accessory interactions might be sub-optimal unless the CFPS reaction is properly supplemented (e.g. disulfide bonding, for example). In addition, once enzyme ‘hits’ are identified, these will likely be produced on a larger scale via cell-based workflows since CFPS manufacturing is cost-prohibitive for recombinant proteins with low toxicity. This is because cell fermentation is still required to generate the cell extract for CFPS but the extract comprises only ~30% of the final CFPS reaction volume; hence, the fixed-cost of fermentation is effectively ‘diluted’ with CFPS and necessitates ~3x protein yield (relative to cellular expression) to restore economic productivity calculations (see Supplementary Fig. S5). Despite these technical concerns, our comparative study of nitrilase screening workflows demonstrates that promising candidates can be rapidly identified using a non-supplemented commercial lysate with minicircle RCA template. For a small number of enzymes, the benefits of our approach may be limited, but if the number of enzymes in a screening campaign becomes large enough, an automated, high-throughput CFPS workflow has the potential to save significant time and labor effort. Further experimentation across a wider set of enzyme classes should be performed to determine the generality of our approach. However, given the inherent unknowns of conventional cell-based screening, CFPS techniques using rolling-circle amplification offer a promising way to accelerate and increase biocatalytic enzyme diversity for industrial applications.

## Materials and methods

### DNA acquisition and preparation of DNA constructs

For cell-based expression, cDNA sequences for the 16 putative nitrilases were ordered from Genscript as pET-24b plasmid constructs (see Supplementary sequence file online). Upon receipt, the cDNAs were also sub-cloned into pEXP5 plasmid (Thermo Fisher Scientific). All enzyme cDNA included an N-terminal 6x His tag within each plasmid or linear gBlock construct. For construction of DNA minicircles from gBlocks (IDT), cDNA sequences were re-coded using a proprietary GE method to optimize for cell-free protein synthesis. Flanking sequences were designed around the sequence-optimized cDNA using the 5′ and 3′ UTRs of the pET-24b control vector. The 5′ UTR flanking sequence of each synthetic gBlock comprised the following sequence elements (listed in 5′ to 3′ order): short leader sequence, BamHI restriction site, T7 pre-promoter sequence, T7 promoter, T7 leader RNA sequence with stem-loop, T7 gene 10 translational enhancer and Shine-Dalgarno sequence. The 3′ UTR flanking sequence for each synthetic gBlock compromised the following sequence elements (listed in 5′ to 3′ order): T7 transcriptional terminator sequence, PstI restriction site, BglII restriction site, and short leader sequence. All synthetic DNA sequences passed IDT gBlock input criteria for custom gene synthesis and were delivered at approximately 1 µg scale.

### Rolling-circle amplification of synthetic DNA fragments and plasmids

For RCA propagation of synthetic DNA fragments, DNA gBlocks were resuspended in TE according to IDT recommendations and approximately 80–100 ng were digested with BamHI and BglII, followed by ligation using T4 DNA ligase, in a thermocycler set to cycle between 7.5 °C and 37 °C at 30 sec intervals to facilitate intramolecular ligation. After 200 cycles, the temperature was raised to 70 °C for 10 minutes to heat-inactivate T4 DNA ligase. Exonuclease I and exonuclease III were then added and incubated at 37 °C for 1 hr to degrade any non-circular DNA, followed by heat-inactivation at 80 °C for 20 min. The resulting DNA minicircles were amplified using a modified illustra^TM^ Single Cell GenomiPhi^TM^ DNA Amplification kit (Cytiva, formerly GE Healthcare Life Sciences) in which the random hexamer primers were optionally replaced with proprietary LNA-containing hexamers and 1-thio-dNTPs were optionally spiked into the Amplification Mix to further thioate the RCA product. While not essential, these modifications tend to improve the DNA concentration of the RCA product and corresponding translational yield in cell-free extract (see Supplementary Fig. S4). Minicircle RCA reactions were incubated isothermally overnight, then heat-inactivated at 60 °C for 20 minutes. The resulting non-purified double-stranded RCA products were quantified using Quant-iT™ dsDNA High-Sensitivity Assay Kit (Thermo Fisher Scientific) in which several 1 µL samplings of minicircle RCA product were measured to normalize for sub-sampling error. Finally, a small portion of each minicircle RCA product was digested with PstI enzyme to confirm accurate restriction fragment analysis by gel electrophoresis. Unpurified minicircle RCA products were subsequently stored at 4 °C until use. Similarly, pEXP5 plasmids were amplified using illustra TempliPhi DNA amplification kit (Cytiva, formerly GE Healthcare Life Sciences) and the unpurified product was stored at 4 °C until use.

### Cell-based protein production

Glycerol stocks of pET-24b nitrilase constructs in *E. coli* BL21 cells were used (10 µL of thawed stock) to inoculate 180 µL of LB + Kanamycin (50 µg/mL) + 1% glucose in Nunc 96-well Flat bottom plates. Inoculums were grown overnight at 30 °C, 85% humidity, 200 rpm and then 15 μL of the saturated culture was used to inoculate 380 µL of TB + Kanamycin (50 µg/mL) in a 2 mL 96-well Deep Well plate. Culture plates were shaken at 30 °C, 85% humidity, 250 rpm for approximately 2.5 h, after which they were induced for overexpression with IPTG (final concentration 1 mM). Inducted plates were shaken for an additional 16–20 h at 30 °C, 85% humidity, 250 rpm, and the cells were subsequently harvested via centrifugation at 4000 rpm for 10 min at 4 °C. The supernatant was discarded, and cell pellets were frozen and stored at −80 °C until ready for use. To prepare the cells for chemical reaction assays, the 96-well Deep well plate containing the putative nitrilase cell pellets were first thawed for approximately 20 min at room temperature. Lysis buffer (200 µL/well, 100 mM potassium phosphate pH 7.0, 1 mg/mL lysozyme, 0.5 mg/mL polymyxin B sulfate, and 2.5 µL benzonase) was added to each well and the plate was shaken at room temperature, 900 rpm for 2 h to resuspend and lyse the cell pellet. The cell debris was pelleted via centrifugation at 4000 rpm for 10 min at 4 °C, and the supernatant was used without further purification in nitrilase reaction assays.

### Cell-free protein production

Unpurified RCA plasmids or minicircle RCA products were directly supplied as template for coupled transcription-translation reactions using the Expressway^TM^ Cell-Free Expression System (Thermo Fisher Scientific, K990097) with minor modification. Briefly, reaction size was scaled down to 50 μL total volume, approximately 0.5 µg of well-mixed DNA was supplied into each reaction, and reactions were incubated at 30 °C in plates with shaking at 300 rpm at 85% humidity. Feed mix was added as prescribed by the manufacturer but in volumes proportionally adjusted to the reaction size. After 6-hr total incubation time, reactions were centrifugated at 4000 rpm for 5 minutes at 4 °C and the supernatant was used to assess enzymatic activity of each translation product.

### Nitrilase activity measurements

Both cell-based and CFPS preparations were assayed to compare putative nitrilase activity on representative substrates. Benzonitrile, cinnamonitrile, 3-phenylpropionitrile, mandelonitrile, and 2-thiopheneacetonitrile were all obtained from Sigma Aldrich. Reactions were assayed under the following conditions: 55 µL of 100 mM KPi buffer pH 7.0, 5 µL of 400 mM nitrilase substrate in DMSO, 40 μL of lysate (either CFPS or cell-based as described above), total reaction volume (100 µL in 96-well Nunc Flat plate). The reaction plates were sealed with a pierceable aluminum seal and incubated for approximately 15 hours at 30 °C and 500 rpm. In order to prepare the plates for analysis, the reactions were diluted with 100 µL of acetonitrile and then sealed and shaken for 40 min at 900 rpm at room temperature. The solid debris was then pelleted via centrifugation at 4000 rpm for 10 min at 4 °C. The clarified supernatant was then diluted 10x into 50:50 water:acetonitrile prior to HPLC analysis. Peak areas were integrated at 230 nm for the nitrilase substrate and hydrolysis product (except for samples containing 3-phenylpropionitrile which were integrated at 210 nm) and nitrilase hydrolysis activity was determined by: peak area hydrolysis product/(peak area hydrolysis product + peak area nitrile) × 100% (see Supplementary Fig. S3 and representative chromatograms in Supplementary Methods).

## Supplementary information

Supplementary Figures and Information.

Supplementary Data.
