# Supplementary material for: Rolling circle amplification of synthetic DNA accelerates biocatalytic determination of enzyme activity relative to conventional methods
Source: Sci Rep. 2020 Jun 24;10:10279. doi: 10.1038/s41598-020-67307-9 (PMC7314814; doi:10.1038/s41598-020-67307-9)

# Rolling circle amplification of synthetic DNA accelerates biocatalytic determination of enzyme activity relative to conventional methods

Timin Hadi[†], Nicole Nozzi[†], Joel O. Melby[†], Wei Gao[‡], Douglas E. Fuerst[†], Erik Kvam[‡*],

[†]GlaxoSmithKline, 1250 South Collegeville Road, Collegeville, Pennsylvania 19426, USA.

[‡]GE Global Research, One Research Circle, Niskayuna, NY 12309, USA.

**Supporting Information**

Supplementary Experimental Methods (pg. 2-3)

Supplementary Table S1 (pg. 4)

Supplementary Figure S1 (pg. 5)

Supplementary Figure S2 (pg. 6)

Supplementary Figure S3 (pg. 7)

Supplementary Figure S4 (pg. 8)

Supplementary Figure S5 (pg. 9)

**UPLC analysis of nitrilase reactions.**

Nitrilase conversion for all substrates was determined using an Agilent 1290 UPLC with a Zorbax SB-C18 RRHD column( 3.0 x 150 mm, 1.8 µm). The column temperature was set to 60 °C, the flow rate set to 1.5 mL/min, and the mobile phases used were 0.05% trifluoroacetic acid in water (A) and 0.05% trifluoroacetic acid in acetonitrile. A wavelength of 230 nm was used to monitor starting material and product, except for hydrocinnamonitrile (3-phenylpropionitrile) and hydrocinnamic acid, which were monitored at 210 nm. See below for gradient conditions.

| Time (min) | Mobile Phase A | Mobile Phase B |
|---|---|---|
| 0 | 80 | 20 |
| 0.7 | 34 | 66 |
| 0.8 | 0 | 100 |
| 1.1 | 80 | 20 |
| 1.5 | 80 | 20 |

**Example Benzonitrile Chromatogram**

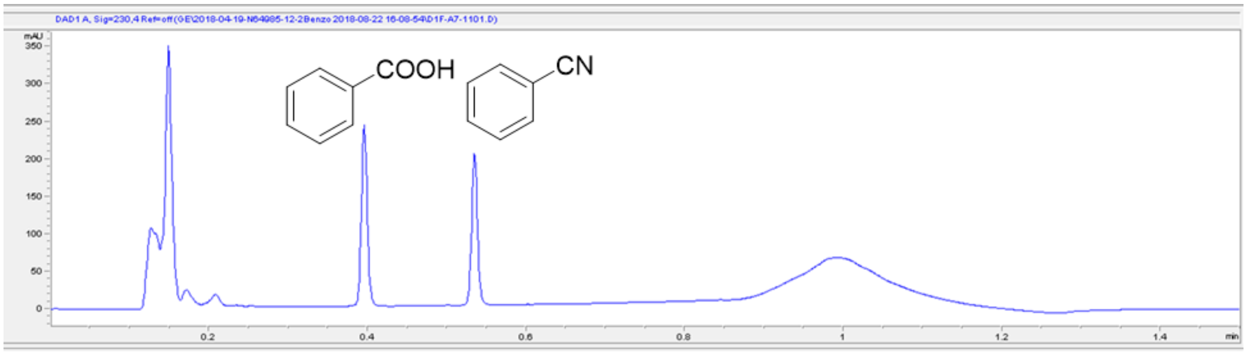


Benzoic acid eluted at 0.405 min, while benzonitrile eluted at 0.535 min.

**Example 2-Thiopheneacetonitrile Chromatogram**

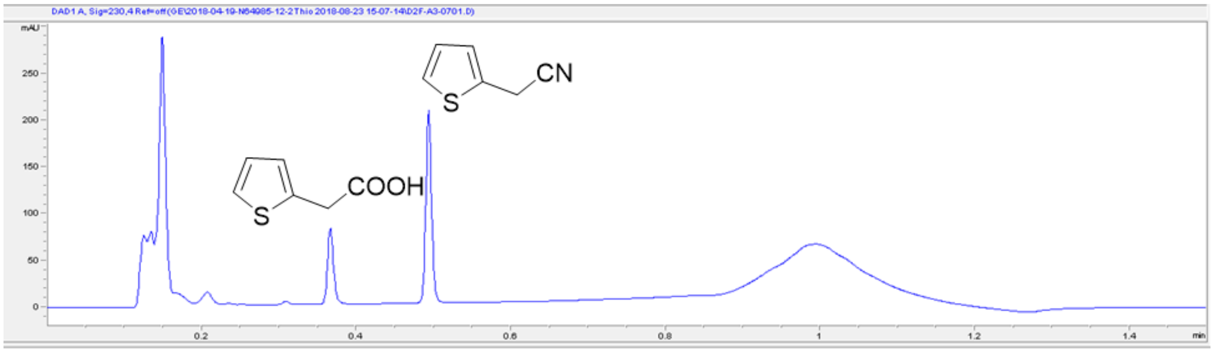


2-Thiopheneacetonoic acid eluted at 0.366 min, while 2-thiopheneacetonitrile eluted at 0.493 min.

**Example Cinnamonitrile Chromatogram**

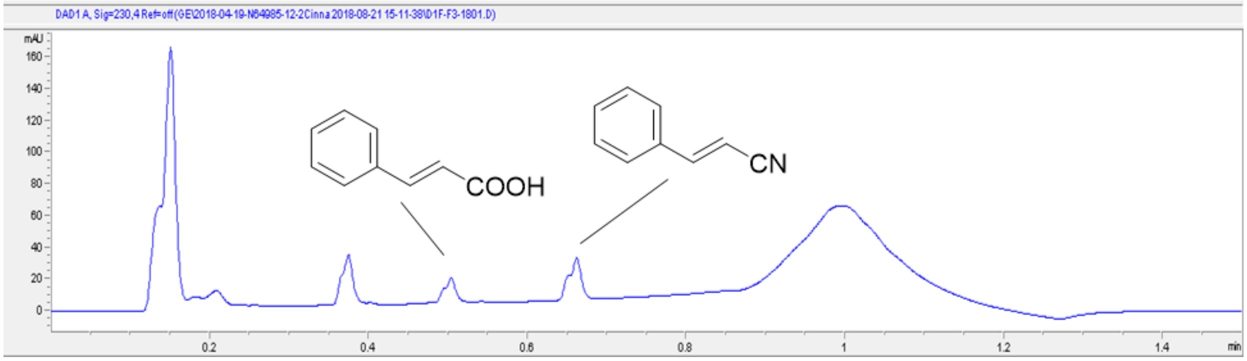


Cinnamic acid eluted at 0.499 min, while cinnamonitrile eluted at 0.659 min. The peak at 0.369 min was presumed to be the primary amide intermediate, but not verified.

**Example Mandelonitrile Chromatogram**

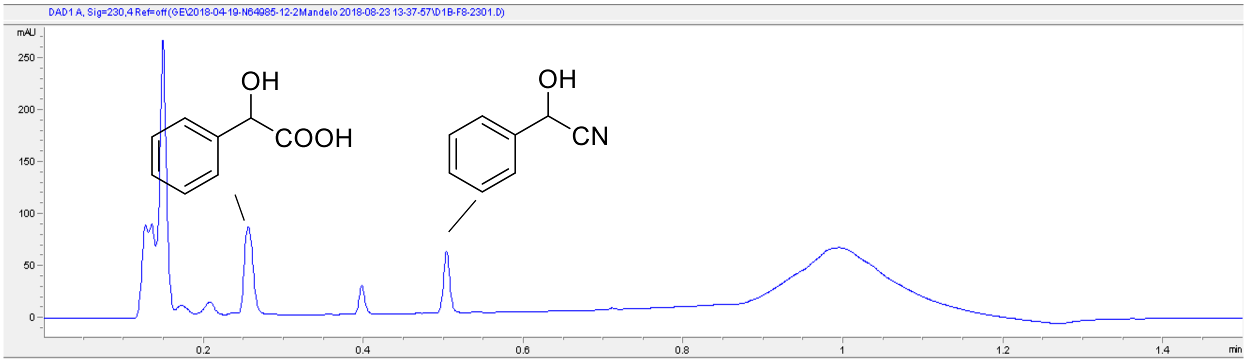


Mandelic acid eluted at 0.256 min, while mandelonitrile eluted at 0.503 min. The peak at 0.397 min was presumed to be the primary amide intermediate, but not verified.

**Example Hydrocinnamonitrile (3-phenylpropionitrile) Chromatogram**

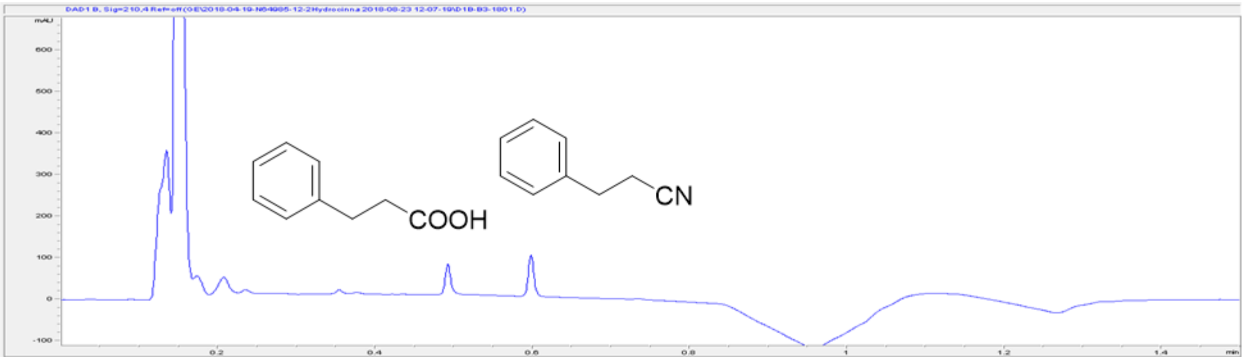


Hydrocinnamonic acid eluted at 0.49 min, while hydrocinnamonitrile eluted at 0.60 min.

**Supplementary Table 1.** List of 16 putative nitrilases including minicircle RCA DNA yields.

| Bacterium |
|---|
| *Acetobacter orientalis* |
| *Halomonas meridiana* |
| *Pseudomonas fluorescens* |
| *Pseudomonas mandelii* |

| Archaeon |
|---|
| *Pyrococcus abyssi* |
| *Thermoplasma acidophilum* |

| Fungus |
|---|
| *Acremonium chrysogenum* |
| *Aspergillus fumigatus* |
| *Aureobasidium melanogenum* |
| *Cylindrobasidium torrendii* |
| *Penicillium expansum* |
| *Penicillium italicum* |
| *Pseudogymnoascus sp.* |

| Nematode |
|---|
| *Dictyocaulus viviparus* |

| Plant |
|---|
| *Gossypium raimondii* |

| ID | GenBank Accession ID | Curated Identity | GE re-coded gBlock size | Minicircle RCA concentration | Minicircle RCA total yield |
|---|---|---|---|---|---|
| Nit1 | WP_010901615 | putative carbon-nitrogen hydrolase (*Thermoplasma acidophilum*) | 1002 bp | 296 ng/μL | 29.6 μg |
| Nit2 | WP_010868514 | nitrilase/apolipoprotein N-acyltransferase (*Pyrococcus abyssi*) | 978 bp | 294 ng/μL | 29.4 μg |
| Nit3 | AAW79573 | NitA arylacetonitrilase (*Pseudomonas fluorescens*) | 1242 bp | 290 ng/μL | 29 μg |
| Nit4 | KEQ58380 | carbon-nitrogen hydrolase (*Aureobasidium melanogenum*) | 1173 bp | 286 ng/μL | 28.6 μg |
| Nit5 | KJB62048 | uncharacterized protein (*Gossypium raimondii*) | 1203 bp | 315 ng/μL | 31.5 μg |
| Nit6 | KEY79035 | nitrilase (*Aspergillus fumigatus*) | 1146 bp | 245 ng/μL | 24.5 μg |
| Nit7 | KJB62045 | uncharacterized protein (*Gossypium raimondii*) | 1377 bp | 253 ng/μL | 25.3 μg |
| Nit8 | GAN66474 | nitrilase (*Acetobacter orientalis*) | 1164 bp | 280 ng/μL | 28 μg |
| Nit9 | KFH48927 | aliphatic nitrilase-like protein (*Acremonium chrysogenum*) | 1218 bp | 286 ng/μL | 28.6 μg |
| Nit10 | KIY65145 | carbon-nitrogen hydrolase (*Cylindrobasidium torrendii*) | 1125 bp | 311 ng/μL | 31.1 μg |
| Nit11 | KFZ01821 | uncharacterized protein (*Pseudogymnoascus spp.*) | 1254 bp | 218 ng/μL | 21.8 μg |
| Nit12 | WP_044630784 | putative carbon-nitrogen hydrolase (*Halomonas meridiana*) | 1116 bp | 191 ng/μL | 19.1 μg |
| Nit13 | KGO60465 | uncharacterized protein (*Penicillium expansum*) | 1170 bp | 202 ng/μL | 20.2 μg |
| Nit14 | KJH52873 | hydrolase, carbon-nitrogen family (*Dictyocaulus viviparous*) | 1191 bp | 205 ng/μL | 20.5 μg |
| Nit15 | KGO74614 | carbon-nitrogen hydrolase (*Penicillium italicum*) | 1131 bp | 202 ng/μL | 20.2 μg |
| Nit16 | AHZ68062 | nitrilase/cyanide hydratase (*Pseudomonas mandelii*) | 1143 bp | 258 ng/μL | 25.8 μg |

**Supplementary Figure 1**. Comparison of benzonitrile hydrolysis activity of 16 nitrilases when produced via minicircle RCA-enabled CFPS or plasmid-based CFPS (pEXP5 propagated by TempliPhi).

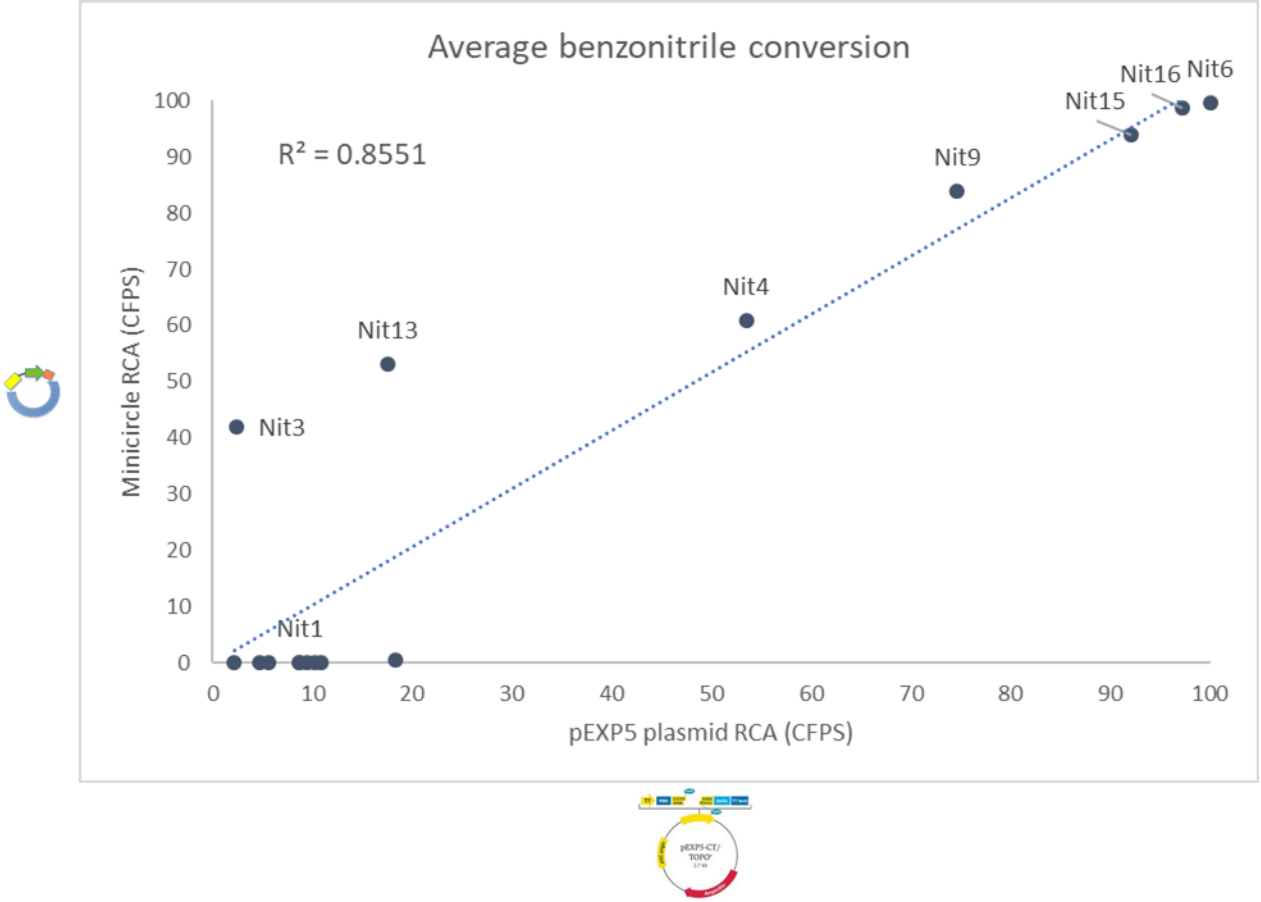

**Supplementary Figure 2**. Effect of DNA concentration on benzonitrile hydrolysis activity from CFPS reactions. “Non-normalized” DNA input contained a fixed template volume (comprising a range of DNA at 715 - 1180 ng) while “normalized” DNA template input was set to 500 ng of DNA.

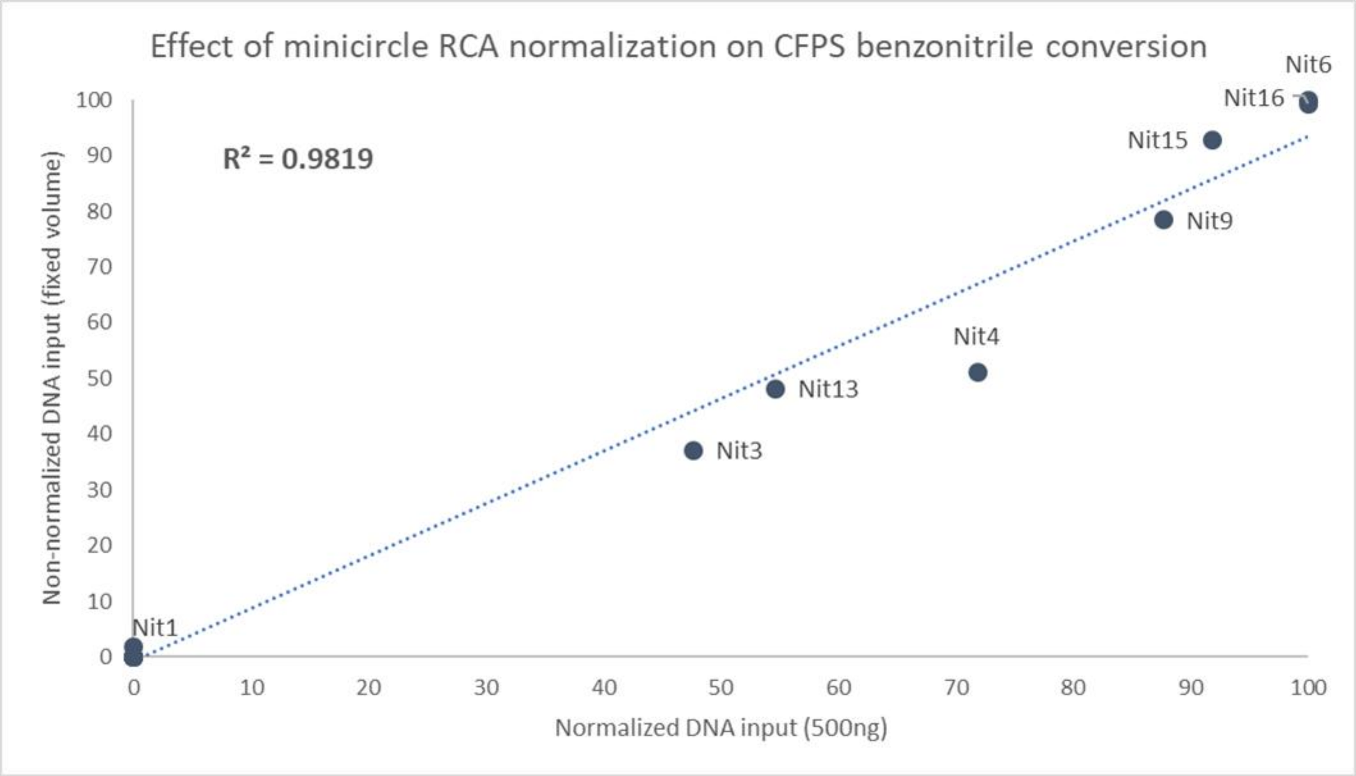

**Supplementary Figure 3.** Average nitrilase substrate hydrolysis across replicate experiments (±SD). Blue bars represent % activity after *E. coli* cell-based expression, while orange bars represent % activity after minicircle RCA-enabled CFPS. Y-axes are percent hydrolysis of the test chemical substrates.

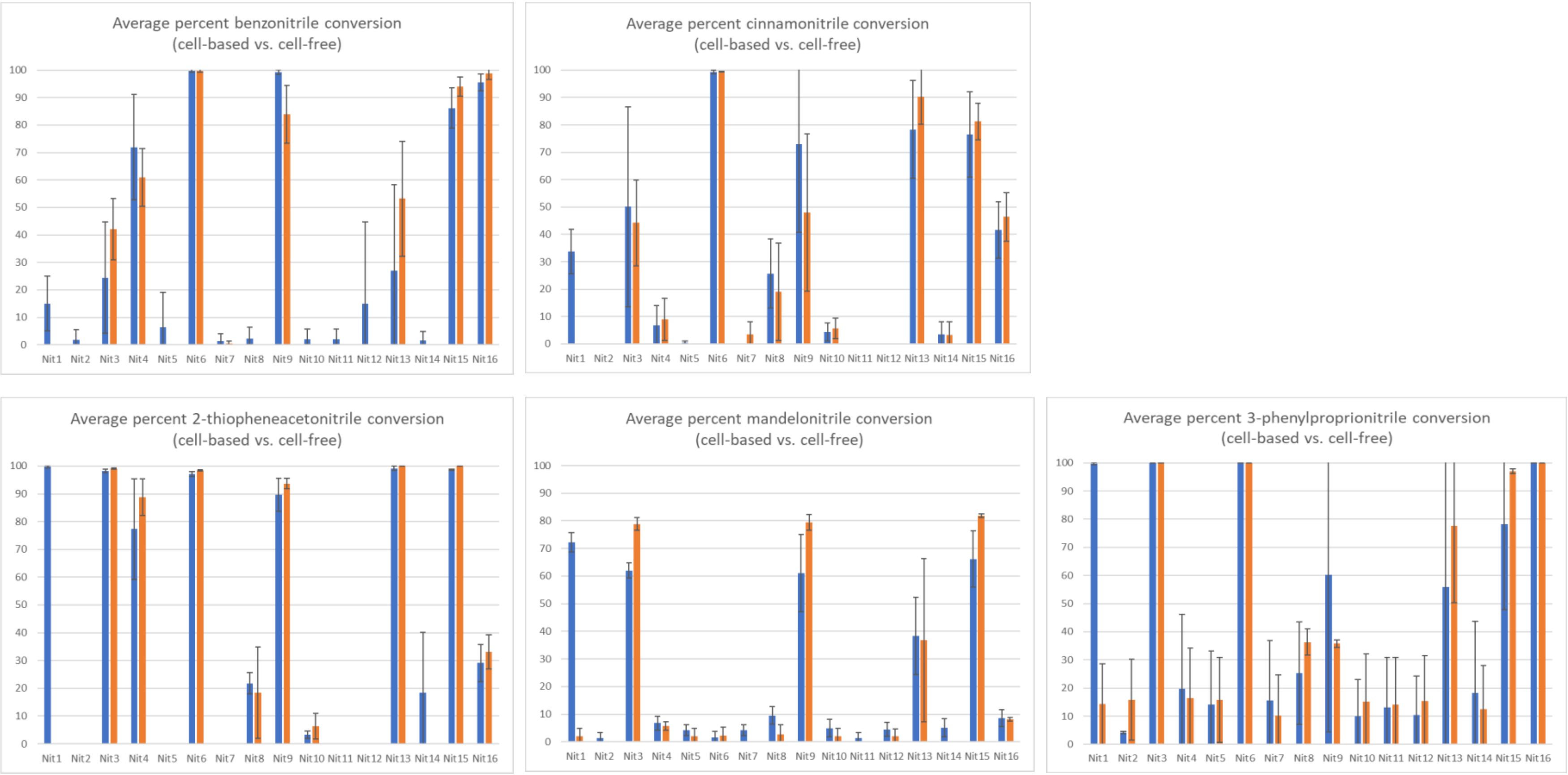

**Supplementary Figure 4.** Comparison of cell-free EGFP yield as a function of minicircle RCA template preparation. Minicircle encoding EGFP was propagated by RCA using either exonuclease-resistant random hexamer (Cytiva, formerly GE Healthcare Life Sciences), or proprietary LNA-containing random hexamer, or exonuclease-resistant random 7mers (Thermo Fisher Scientific, #SO181). Additionally, DNA was further thioated during amplification (where indicated) by spiking $\alpha$-S-dATP into the dNTP mix (to a final concentration of 10 μM). Approximately 0.5μg of minicircle RCA underwent coupled transcription-translation using the ExpressWay system, and fluorescent EGFP product yield was measured at 488nm ex/520nm emi against a purified EGFP standard curve (BioVision, #4999).

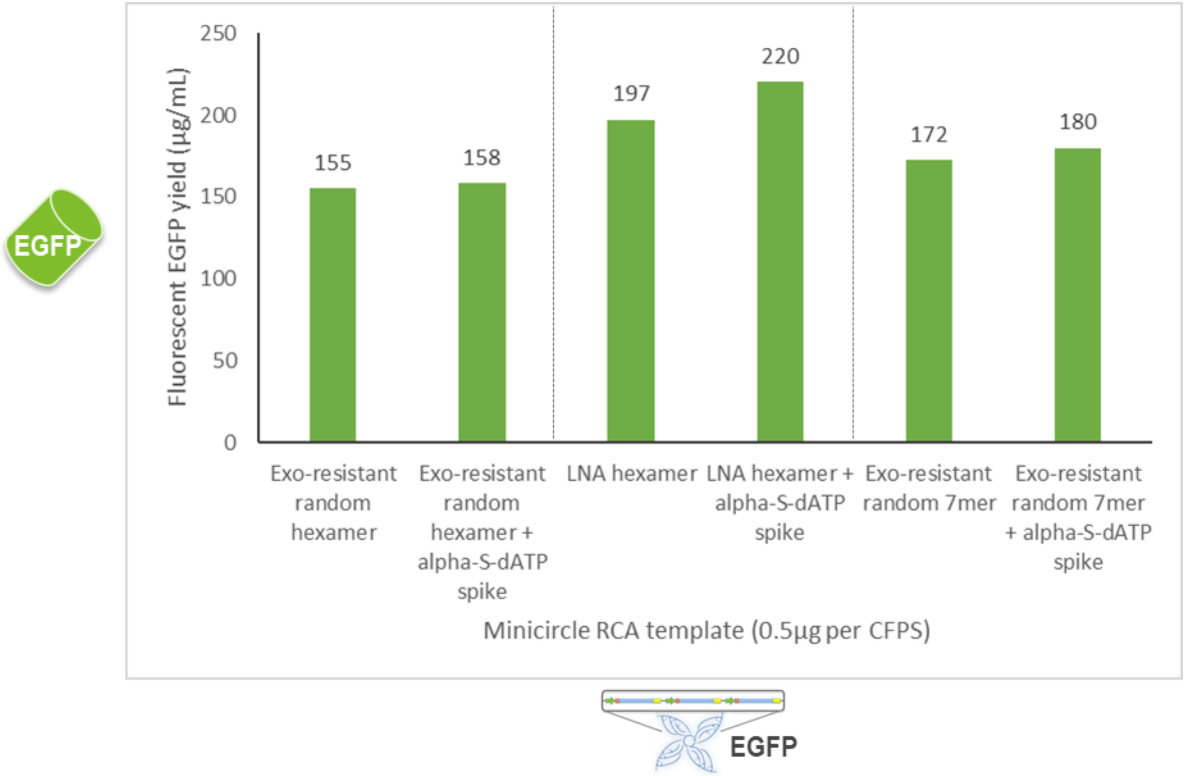

**Supplementary Figure 5.** Hypothetical comparison of cell-based vs. CFPS production of protein-of-interest (POI). A fixed-cost for fermentation is estimated and propagated as a function of POI yield scenarios.

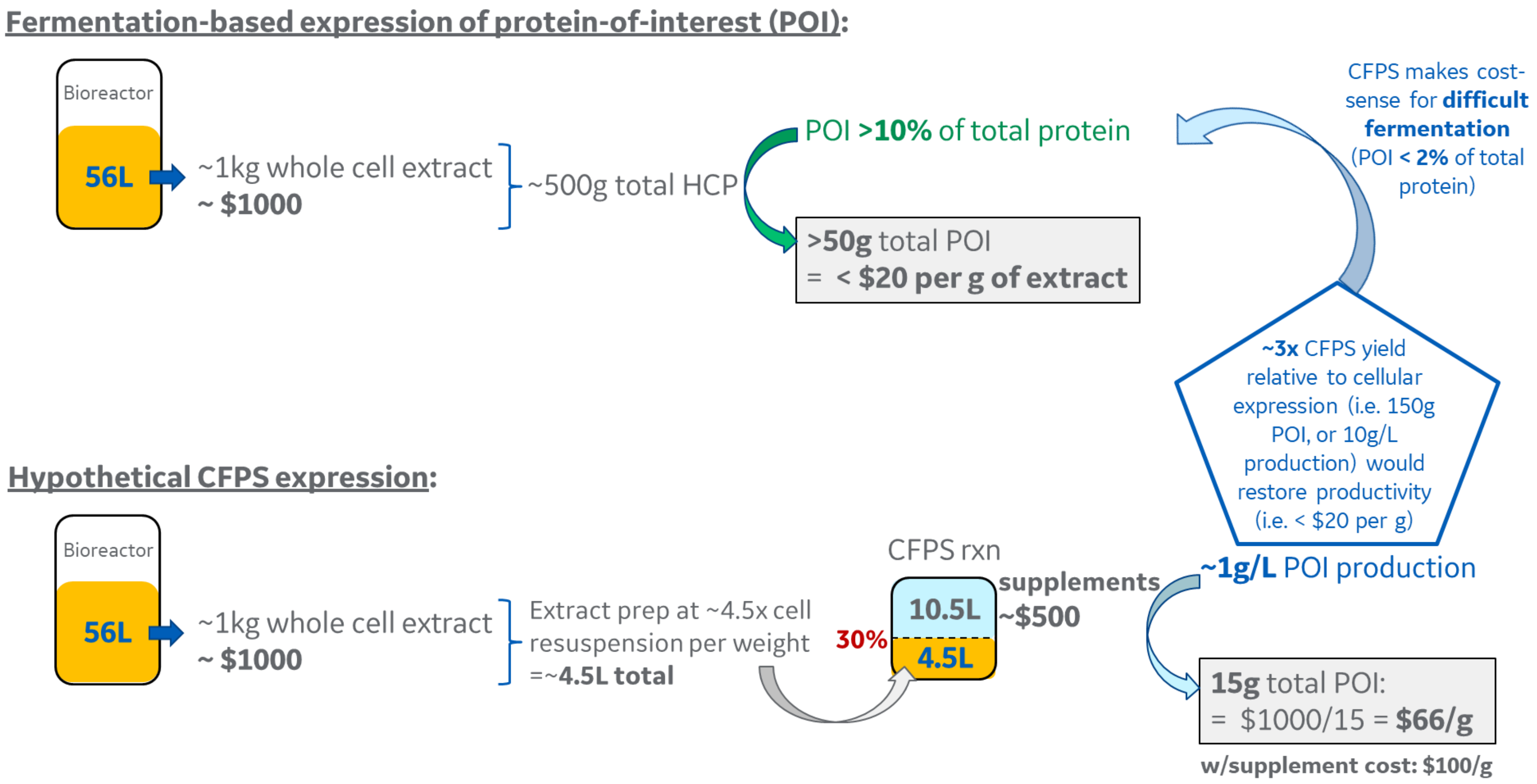

Supplement: Supplementary file 1 — Supplementary Figures and Information. [file 41598_2020_67307_MOESM1_ESM.pdf]
